# Philosophical Perspectives on Doping Sanctions and Young Athletes

**DOI:** 10.3389/fspor.2022.841033

**Published:** 2022-03-18

**Authors:** Sarah Teetzel

**Affiliations:** Faculty of Kinesiology and Recreation Management, University of Manitoba, Winnipeg, MB, Canada

**Keywords:** youth doping, doping sanctions, minors, privacy, autonomy, children in sport

## Introduction

Several recent legal and ethical analyses of drug testing in sport (e.g., McNamee and Møller, 2011; Veber, 2014; Dimeo and Møller, 2018; Murray, 2018; Haas and Healy, 2019) build on the foundational philosophical arguments presented by Brown (1984), Fraleigh (1984), and Schneider and Butcher (2001). However, these cogent analyses largely focus on autonomous adults who freely choose to participate in sport knowing what the anti-doping system requires of them. Whether young people who break the rules outlined in the World Anti-Doping Code can and should be treated any differently than their adult peers and competitors is unclear. A vast body of research literature confirms that the use of performance-enhancing substances and methods extends far beyond the scope of high-performance sport, and many non-athletic populations regularly use performance-enhancing substances and methods for alternative purposes (Katims and Zapata, 1993; Yesalis and Bahrke, 2000; Miller et al., 2002; Lorente and Grelot, 2003; Laure and Binsinger, 2007; Muller et al., 2009; Ntoumanis et al., 2014; Andreasson and Johansson, 2021; Gleaves et al., 2021). For over two decades, research teams have reported that doping is not restricted to adult athletes (Komoroski and Rickert, 1992; Melia et al., 1996; Goulet et al., 2002; McNamee, 2009; de Hon et al., 2015). These studies highlight and confirm that some young people engage in doping practices, and doping is not restricted to adult athlete populations.

Media coverage of young people committing anti-doping rule violations is also increasingly common. For example, two anti-doping rule violations occurred at the first Youth Olympic Games (YOG), held in August 2010 in Singapore. At the YOG, which currently feature athletes between the ages of 15 and 18, all medal winners as well as randomly selected athletes are required to undergo doping control procedures. At the inaugural YOG, two 17-year-old wrestlers returned positive doping tests, were disqualified, and were required to return their participation certificates and the medal one athlete was initially awarded (Associated Press, 2010). Both were suspended from sport for 2 years, and their names were entered into the public doping registry of the Fédération Internationale des Luttes Associées (FILA; now known as United World Wrestling) despite their status as legal minors (FILA, 2010).

The Olympic movement has long held the position that doping rules are firm, and an athlete's age is irrelevant. This stance is best illustrated with the case of 16-year-old Romanian artistic gymnast Andreea Răducan losing the gold medal at the Sydney 2000 Olympics over taking cold medicine given to her by her team physician. Her appeal to the Court of Arbitration for Sport's *ad hoc* division, on the basis she was not responsible for the anti-doping violation, was unsuccessful. Arbitrators ruled that her status as a minor did not negate the fact a banned substance was found in her urine sample (Teetzel and Mazzucco, 2014). IOC Executive Board member, Dr. Jacques Rogge, who was appointed president of the IOC a year later, acknowledged to reporters the injustice of the situation: “This is one of the worst experiences I have had in my Olympic life. Having to strip the gold medal from the individual gymnastic champion for something she did not intentionally do is very tough. But the rules are the rules” (BBC Sport Online, 2000). The Court of Arbitration for Sport's acting secretary general at the time, Matthieu Reeb, also acknowledged the injustice, noting, “The panel is aware of the impact of its decision on a fine, young, elite athlete” (BBC Sport Online, 2000). Răducan's disqualification aligned with a decision at the 1972 Summer Olympics to disqualify 16-year-old swimmer Rick DeMont of the USA following his gold medal performance in the 400 m freestyle, after his doping control sample tested positive for ephedrine. All involved in the case understood that the ephedrine found in his sample came from his prescription asthma medication, and that his team physician had made an error in not disclosing the athlete's required use of the medication, yet the disqualification stood (Hunt, 2011).

Concerns about youth doping were amplified at the 2022 Olympic Winter Games in Beijing following the news between the team figure skating finals and scheduled medal award ceremony that 15-year-old figure skater Kamila Valieva, representing the Russian Olympic Committee, had tested positive for the banned substance trimetazidine. The delayed news of her positive test, which was collected on December 25, 2021 at the Russian Figure Skating Championships in Saint Petersburg, but not released until February 8, 2022, the day after the team figure skating finals, raised many questions and resulted in considerable speculation. Despite objections from the World Anti-Doping Agency, International Olympic Committee, and the International Skating Union, the Russian Anti-Doping Agency (RUSADA) Disciplinary Committee lifted the mandatory provisional suspension applied to Valieva and argued before an emergency meeting of the *ad hoc* division of the Court of Arbitration for Sport that Valieva should be permitted to continue competing at the Winter Olympics in the upcoming women's event. WADA quickly clarified in a press release that the World Anti-Doping Code does not allow exemptions from mandatory provisional suspensions for anyone, including minors who, like Valieva, fall under the relatively new category of “protected persons” (WADA, 2022). As per the WADA Code, WADA defines protected persons as athletes who are under 16 years (or under 18 if the athlete is not part of a registered testing pool or competed at international events) or “for reasons other than age has been determined to lack legal capacity under applicable national legislations” (WADA, 2021, p. 174). The Code also stipulates that mandatory public disclosure is not required when a protected person commits an anti-doping rule violation, but it does not prohibit media reporting on the athlete. Specifically, the Code notes, “Any optional Public Disclosure in a case involving a Minor, Protected Person or Recreational Athlete shall be proportionate to the facts and circumstances of the case” (WADA, 2021, p. 102).

With the introduction of the protected persons category in recent years, WADA has recognized that young athletes require protection. Indeed, much of the resulting vitriol and outrage at the decision to allow Valieva to continue competing at the Winter Games, pending her B sample results and appeal process, was directed at her controversial coach, Eteri Tutberidze, and the Russian sport system, generally, not at the 15-year-old athlete herself. Consensus emerged quickly that the adults influencing and controlling Valieva were the ones to blame. Unlike with past cases, more people are recognizing that youth cannot fully comprehend the implications of taking a banned substance or providing a doping sample, particularly if coercion or parental pressure is involved. To highlight the additional ethical concerns that are present when young athletes commit anti-doping rule violations, this paper examines the concepts of childhood, autonomy, and privacy from a philosophical perspective focusing on how each relates to young athletes who dope. Conceptual clarification at the metaphysical level is always beneficial before attempting to address any ethical issue in sport (Kretchmar, 1983). After analyzing how influential conceptions of childhood, privacy, and autonomy apply to young athletes, I argue that legitimate expectations of privacy and autonomy in the context of doping are not being recognized in sport. This vulnerable athletic population, by definition, has not developed the capacity to make rational, independent decisions and therefore should not be held to the same level of fault or face the same consequences as adult athletes who commit anti-doping rule violations. In summary, age ought to matter more than it currently does.

## Special Considerations of Childhood

The American Academy of Pediatrics' (2005) policy on performance-enhancing drugs acknowledges that children are the most vulnerable population affected by doping. However, children have not always been considered such a vulnerable population, in sport or in everyday life. As the history and philosophy of childhood literature establishes, the division between childhood and adulthood, and the time in between these descriptors, is hard to categorize and is culturally conditioned (Cole and Cole, 1996; Schapiro, 1999; Matthews and Mullin, 2020). Developmental psychologists recognize adolescence as the transitory period between childhood and adulthood where individuals reach a level of maturity and personal identity. However, as Neil Postman's work on the history of childhood demonstrates, the view of childhood as a social structure dates back only to the sixteenth century. Arguing that increased literacy associated with the Renaissance led to the recognition children require protection stemmed from conceptualizing the “adult world” as one that excluded children. With this recognition, people had to develop skills and “earn” adulthood, not just grow older; as a result, illiterate older people began to be grouped with children in the category of non-adults. Literacy and education motivated societal understanding of the importance of childhood when adulthood came to be marked by “the requirements of a fully literate culture: the capacity for self-restraint, a tolerance for delayed gratification, a sophisticated ability to think conceptually and sequentially, a preoccupation with both historical continuity and the future, a high valuation of reason and hierarchical order” (Postman, 1994, p. 99).

Fast forwarding to the Victorian era, the concept of childhood evolved to be considered a time of innocence protected from the demands of labor (Mayall, 2000). Despite that recognition, a clear distinction between childhood and adulthood has not ever been fully embraced in the literature, in public policy, or around the world, and the terms “youths,” “minors”, and “adolescents” continue to be used interchangeably to describe the period between childhood and adulthood. For example, youth as per the Youth Olympic Games eligibility criteria, are people aged 15-18. The United Nations (UN) uses age 18 as the start of adulthood, stipulating in Article 1 of the Convention on the Rights of the Child that children represent “every human being below the age of 18 years unless under the law applicable to the child, majority is attained earlier” (UN, 1989). In addition, the United Nations consider “youth” to be people aged 15–24, recognizing member states may apply different definitions or age ranges for individuals considered to be youth (UN, 2021). WADA sets the limits of 16 years of age for “protected persons” in most situations, but also clarifies “minors” otherwise refers to people under 18 years of age (WADA, 2021, pp. 171, 174).

The arbitrariness of these definitions is clear in sport. While the Convention on the Rights of the Child suggests age limits for engaging in paid labor, it does not address elite sport participation and training (Farstad, 2007). Moreover, international standards addressing children's rights are not adopted by all governments, making compliance unenforceable globally (Mazzucco, 2012). Similarly, protections that stem from research ethics boards with respect to children's involvement in non-therapeutic research do not extend into completion and training for sport either (Schneider and Butcher, 2001). As a result, child labor, child trafficking for sport, and the treatment of child athletes are among the most pressing issues in sport (Tymowski, 2001; Grenfell and Rinehart, 2003; Donnelly, 2008). Outside of sport, children are considered a vulnerable population requiring care and consideration to ensure they are protected from harm. However, a substantial number of young athletes have risen to the top of their sports and achieved remarkable success quite early in their lives and careers. For example, using the UN recommendations, diver Fu Mingxia of China was a child when she won the 10 m platform diving event at the Barcelona 1992 Summer Olympics at age 13, and much further back Aileen Riggen was 14 years old when she won the women's diving event at the 1920 Olympics in Antwerp, while figure skater Sonja Henie was a mere 11-year-old child at the time of her Olympic debut. Romanian gymnast Nadia Comaneci was 14 when she achieved the first perfect 10 in Olympic gymnastics, and swimming sensation Michael Phelps was 15 when he competed in his first Olympic Games in 2000 and set his first world record in the 200m butterfly. These legendary Olympians were children at the time of their success according to most countries' laws. In terms of physical, intellectual, and moral development, these young athletes were girls and boys, not women and men. However, complicating the distinction between child and adult, children mature at different rates and some 14-year-olds, for example, might be more mature than an immature 20-year-old opponent.

With recognition of the arbitrariness of doing so, this paper focuses on young athletes in the age range 15–17 who are developing the ability to make decisions in their own best interests. These athletes are eligible to compete at the YOG (pending their IF not stipulating a different age range within the 15–18 range, as the IOC permits IFs to do) yet fully within the UN's definitions of children and youth. While resisting the idea that one's 18th birthday magically makes a person a competent adult, in this paper the legal age of adulthood in most countries is used as the onset of adulthood, and adult athletes are considered in what follows as athletes who are 18 years of age and older.

## Philosophical Conceptions of Autonomy

Discussions of autonomy in sport are prevalent with respect to participants' ability to consent to participate in the so-called violent sports, or in activities like cockfighting and rodeo (Dixon, 2016). Other areas where arguments from autonomy feature prevalently relate to risk and athletes' decisions to engage in risky recreational pursuits like BASE jumping and big wave surfing (Creyer et al., 2003). When arguing in favor of eliminating the current doping bans in elite sport, some of the most convincing arguments used by sport philosophers, including Brown (1984) and Tamburrini (2000) appeal to athletes' autonomy and right to make independent decisions about matters pertaining to their bodies. However, the legitimacy of the restrictions outlined in WADA's World Anti-Doping Code are also based on athletes' autonomy, but in this case their abilities to choose to voluntarily accept the conditions of participating, including that they cannot consume or use banned substances or methods without consequences. On both sides of the issue, the philosophical concept of autonomy features prominently in guiding our collective thoughts on moral issues in sport. The idea of autonomy as self-rule has been debated by philosophers for centuries and described in numerous ways; as a result, myriad conceptions of autonomy can be found in the philosophy literature. Regardless of whose definition is used, there are commonalities among what we mean when we declare someone is or is not autonomous, which matter in evaluating youth athletes' abilities to make independent decisions in sport.

Western historical sources trace discussions of autonomy back to the Ancient Greeks, referring often to the self-rule enjoyed by several Greek city-states, not by individuals. The Ancient Greek origin is evident in the word itself, which divided into its roots, *Autos* (self) and *Nomos* (meaning rule, governance, and law), results in the concept of self-rule. A consensus on what the idea of “self-rule” really means today in the twenty-first century is not as clear. An autonomous individual is often defined as a person whose moral principles are one's own; however, this does not tell us what autonomy is or why we should value it and work to protect it, particularly in sport. The view that autonomy is a necessary value is found in many foundational ethical theories, including Kant's (1785/1983) moral philosophy as well as Mill's (1859/1975) liberal utilitarianism, and autonomy is often a central virtue in both virtue ethics and ethics of care (Christman, 2020). Autonomy can be conceived of as a moral, political, and social ideal to make sense of intuitions and normative claims (Dworkin, 1988). The concept is always context dependent, lacks an essential definition, and may not be met with universal approval.

One reason autonomy is difficult to define is its similarities with other philosophical concepts, such as dignity, freedom, independence, individuality, integrity, responsibility, self-determination, and sovereignty. Other descriptors connected to autonomy highlight the connection between autonomy and independent action, including the ability do as we please, the capability to intentionally self-initiate actions, freedom from obligations imposed by others, and the absence of coercion, deception, or force. Finally, kindred concepts related to self-reflection and self-knowledge connect deeply to many definitions of autonomy, which include the capacity to make decisions rationally and freely, awareness of your own best interests, and the voluntary pursuit of projects that form your identity. Together these positively and negatively defined descriptors characterize people who act autonomously.

Berlin's (1969) influential essays on positive and negative liberty attempt to answer two central questions: (1) in what area(s) should people be left without interference by others? and (2) how far can government(s) interfere with individuals? Berlin argued that freedom is obedience to a law that one prescribes to oneself, noting: “I am free because, and in so far as, I am autonomous. I obey laws, but I have imposed them on, or found them in, my uncoerced self” (1969, p. 136). These ideas surrounding a necessary recognition of an uncoerced self are present in subsequent analyses of autonomy that emphasize critical self-reflection. For example, according to Dworkin, autonomy is:

A second-order capacity of persons to reflect critically upon their first-order preferences, desires, wishes, and so forth and the capacity to accept or attempt to change these in light of higher-order preferences and values. By exercising such a capacity, persons define their nature, give meaning and coherence to their lives and take responsibility for the kind of person they are (1988, p. 20).

What Dworkin refers to as “second-order capacities” can be understood as our preferences about our preferences, or the ability to think about our reasons for holding a certain preference that we hold. Similarly, “first-order preferences” are those at the most basic level, which are similar to instincts, such as obtaining food and water, whereas “higher-order preferences and values” are those that align with one's principles rather than one's whims or immediate needs. The ability to perform critical self-reflection to recognize your higher-order preferences is an important component of being an autonomous person and making autonomous decisions.

McLeod (2005) summarizes some of the intricacies in discussing the concept of autonomy, arguing:

Autonomy is mostly a philosophical term of art, one that philosophers use in a myriad of ways…(none of us grew up with it surely, unless perhaps we are children of philosophers) we have no pre-theoretical intuitions with which to evaluate how philosophers use it, or so some might claim. But would one be right? I do not think so. ‘Autonomy' represents a phenomenon with which people do have some experience and on which they could comment in a pre-theoretical way. The phenomenon itself is that of self-government or self-rule, as opposed to government or rule by others (p. 1).

With respect to sport and athlete autonomy, McLeod's succinct definition, “When we govern our own actions and choices we are autonomous; when someone else does so, we are not” (p. 10) is helpful. Feminist views of autonomy acknowledge that autonomy has practical value in understanding gender oppression and objectification (Govier, 1993), while recognizing that oppressive practices undermine and diminish a person's autonomy (Stoljar, 2018).

Bioethicists Tom Beauchamp and James Childress's identification of respect for autonomy as a core principle of biomedical ethics emphasizes the importance of autonomy in matters pertaining to our health and wellness. Their understanding of autonomy as a core principle includes the idea that a practical understanding of autonomy is “not excessively individualistic, not excessively focused on reason (neglecting the emotions), and not unduly legalistic (highlighting legal rights and downplaying social practices)” (2001, p. 57). In an applied ethics setting, Beauchamp and Childress advocate analyzing autonomous actions by whether an individual can make decisions and act intentionally, with understanding, and without any controlling influence. What is relevant here with respect to youth doping is whether youth athletes can undergo this type of reflection, free of coercion from those invested in their athletic success.

Applying the concept of autonomy in sport requires the blending together of the elements of liberty, independence, and critical self-reflection that can be found in the philosophical works described above. Acting paternalistically often involves violating other people's autonomy by seizing their decision-making powers, which is often the case when a parent or coach acts on behalf of a young athlete (Dixon, 2007). Examples include choosing a sport for a youth to specialize in or deciding how many hours per week youth athletes will train. A recurring theme in the philosophical literature on both autonomy and privacy is the uncertainty regarding how much of each a person can expect and demand.

## A Right to Privacy?

Privacy discussions in the law and ethics literature often start with Warren and Brandeis's (1890) definition of privacy as “the right to be let alone.” Since then, the difficulties involved in producing a universally accepted definition of privacy have led many philosophers to acknowledge that a precise definition may be impossible because of the lack of consensus on both if and why we may have a right to privacy (Alfino and Mayes, 2003). Given the differing emphasis on privacy around the globe and among different cultures, debates continue on whether privacy ought to be considered a basic human right.

Putting aside the question of privacy's inclusion on the list of human rights, privacy is valuable for several reasons, including that “it protects what people deem important in life, such as the intimate sphere or the conditions for autonomous judgment” (Beckman, 2005, p. 98). Privacy includes a measure of protection:

Privacy shields us not only from interference and pressures that preclude self-expression and the development of relationships, but also from intrusions and pressures arising from others' access to our persons and the details about us. Threats of information leaks as well as threats of control over our bodies, our activities, and our power to make our own choices give rise to fears that we are being scrutinized, judged, ridiculed, pressured, coerced, or otherwise taken advantage of by others…Loss of privacy leaves us vulnerable and threatened (DeCew, 1999, p. 249).

The threat of constant observation by others can cause people to censor their movements and behaviors, which WADA's whereabouts program draws on as a component of the anti-doping system in sport. In sport, discussions of privacy and autonomy often intersect.

The link between respecting privacy and treating people as autonomous beings worthy of respect is a central principal of bioethics, with many recognizing privacy as a moral rule necessary for researchers and medical professionals to respect (Beauchamp and Childress, 2001). Violating a person's privacy denies that person the power to control who has access to privileged information about their self and body. Children are frequently denied this right, which is instead bestowed upon their legal guardian(s) tasked with making decisions for them in their best interest with the intent of ensuring they maintain an open future (Miah and Rich, 2006). As children mature at different rates, it is difficult to pinpoint the time that young athletes should be entitled to the same expectations of privacy that adults receive. Some critics of the current doping policies and rules note that the whereabouts program requirements and sample collection methods impinge upon athletes' right to privacy.

The Privacy Commissioner of Canada in a report on drug testing and privacy bluntly acknowledged that drug testing is an invasion of privacy. Specifically, the report noted: “the principal privacy issue flowing from drug testing is not whether testing is intrusive. It is. Urinalysis is particularly intrusive, requiring as it may either a pre-test physical search, the direct observation of an intimate bodily function, or both. The principal issue is in what circumstances the intrusions occasioned by testing are justified” (Privacy Commissioner of Canada, 1990, p. 22). In Canada, the privacy officer deems drug testing to be defensible on utilitarian grounds when public interest is at stake, explaining, “while there is no doubt that drug testing infringes personal privacy in a profound sense, one must not be blind to the need to protect the public interest” (p. 3). Public safety supersedes individual privacy for pilots, corrections officers, and some medical personnel, as people in these occupations are subject to drug testing as a condition of their employment. But the same argument is weak when applied to sport. The right to privacy is made more complex when genetic information is at stake (Privacy Commissioner of Canada, 1992). How children fit into the equation is not addressed; yet this problem is magnified when the athletes in question are considered children incapable of making decisions of this nature. In the context of doping, detection tests require athletes to consent to provide access to their personal information via the “data” contained in their blood and urine even though accessing this type of information violates privacy rights in many areas of the world (Teetzel, 2007).

## Ethical Considerations in Youth Athletes' Rights to Autonomy and Privacy in the Context of Doping in Sport

When young athletes use banned substances or methods to increase their performance, additional ethical concerns arise beyond those associated with adult doping (McNamee, 2009; Mountjoy et al., 2015). Most obviously, the stigma of a positive doping conviction can haunt young athletes for the rest of their careers, and even their lives. For example, when Polish athlete Igor Walilko tested positive for nikethamide and received an anti-doping rule violation and subsequent 2-year ban from the Federation Internationale de L'Automobile, his family hired a lawyer to challenge the decision. As Walilko was only 12 years old at the time of the incident, his lawyer argued he could not be considered criminally liable for doping as he was too young to even compete at the Youth Olympic Games (Carmichael, 2011). Despite his age, and the banned substance being traced back to an energy bar, the Court of Arbitration for Sport only recommended reducing his WADA-imposed ban from 2 years to 18 months, calling it “excessive and disproportionate” but agreeing the athlete was not too young for the anti-doping rules to apply. Accordingly, his lawyer noted, “He was very famous in Poland and, 1 day after, he was a criminal child” (Hyde, 2011).

Long-term stigmas and lifelong repercussions can impact any athlete found to have cheated with banned substances or methods. For example, after Ben Johnson tested positive for an anabolic steroid at the 1988 Olympic Games, his reputation never recovered, and his name remains synonymous with cheating and doping in sport. Johnson was 27 years old when he was caught doping in Seoul, but his use of anabolic steroids is thought to have started much earlier, and his disqualification has negatively impacted his subsequent opportunities for employment and sponsorship. Unable to capitalize on his image or attract new sponsorship opportunities, years later Johnson agreed to be featured in a television commercial for the sports drink Cheetah Power Surge where he participated in a staged race against a cheetah, and responded to the question, “Ben, when you run, do you Cheetah?” emphatically noting, “Absolutely, I Cheetah all the time.” The ad ends with him recommending to consumers to “go ahead and Cheetah” (YouTube, 2012). Johnson serves as a cautionary tale to athletes considering using banned substances or methods (Moore, 2012). The duration of the shame, and onset during the time an adolescent is maturing and entering adulthood, may have even more lasting consequences on self-image and future prospects.

Paternalistic interventions in sport are accepted in recognition of children and youth's vulnerability and susceptibility to coercion and exploitation, with recognition that young athletes are not yet mature legally, morally, or physically (Tymowski, 2000). As Gabriela Tymowski argues, “the moral responsibility ought to be on adults to protect children rather than on children to be precocious in the ways of the world before they are truly ready to meet those challenges” (2000, p. 81). The rules governing participation in high-performance sports allow national and international anti-doping agencies to test athletes competing under their jurisdiction for the use of performance-enhancing substances or methods. Athletes selected for tests must declare their whereabouts, submit to the testing, and provide the requested blood or urine sample under observation. Refusing to do so is taken as an admission of guilt and an anti-doping rule violation. It is easy to see that there is no room to opt out of taking a doping test on the grounds that doing so constitutes an invasion of privacy. Advocates of privacy rights might maintain that this system does not respect the privacy that athletes, as human beings, are entitled to receive, but this claim is contestable as no one is forced to participate in sport at the high-performance level (Kayser et al., 2007).

No matter their age, athletes are prohibited from using substances banned by the World Anti-Doping Code, and tests are needed to ensure anti-doping rules are followed. This creates a problem for respecting autonomy and privacy rights because current tests to detect doping violate youth athletes' justifiable expectations of privacy and autonomy, and it is unclear if they can consent to this violation. This problem is magnified when the athletes in question are youths because it is not obvious to what extent the notions of autonomy and privacy apply to people who have yet to reach adulthood. Legal guardian consent to analyze a youth's blood or urine is sufficient in the context of health and medicine when a person's life or wellbeing is at stake, but sport is a voluntary pursuit. Arguments that support paternalistic decision making in the best interest of the child do not seem as effective in sport as they do in healthcare examples.

The degree of privacy and autonomy a young athlete can expect in the sporting world is debatable due to the prerequisite conditions sport-governing organizations require athletes to adhere to in order to opt in to participate. Parents and coaches make the majority of decisions for young athletes because, in the majority of societies, youth are not considered able to adequately foresee the consequences of their behaviors and make truly informed choices until they reach a level of maturity. Doping control procedures utilized in sport are justified on utilitarian grounds that a “clean” sport system outweighs any indignities providing a sample produces, and that athletes voluntarily agree to participate in this system in order to ensure their competitors compete fairly (Schneider, 1993). The challenges that athletes face as a result of drug testing programs in sport create an interesting case study to analyze the different societal expectations placed on youth athletes in their roles as athletes compared to their entitlements as children.

The sociohistorical literature that addresses youth and doping often returns to the experimental doping studies undertaken during the Cold War, which subjected a large but unknown number of young people to untested drugs, particularly anabolic androgenic steroids, to gain insight into how to enhance performance with drugs. In most cases, athletes did not consent to their inclusion in these experiments, and many faced long-term negative consequences from their forced participation (Dimeo and Hunt, 2012). While public opinion continues to associate the era of state sponsored systemic doping with the German Democratic Republic (East Germany), teenaged athletes from many countries were required to take part in similar experimental protocols (Franke and Berendonk, 1997). Translated documents from the former Soviet Union suggest that adult coaches, trainers, and medical researchers provided performance-enhancing drugs to children as young as seven and eight years old who showed promising athletic potential (Waddington and Smith, 2009).

Beyond steroids, documented cases exist of adults intentionally doping minors with human growth hormone, as well as laxatives and diuretics, to either accelerate growth or delay the onset of puberty in athletes competing in the aesthetic sports. Joan Ryan, known for her advocacy for safe sport, quotes a gymnastics coach explaining the necessity to delay puberty in girls to facilitate athletes maintaining the desired physique, noting: “gymnasts don't so much retire as expire” (Ryan, 1995, p. 34). As a result of investigations like Ryan's, the American Academy of Pediatrics. (2005) acknowledged that consumption of these drugs and others by healthy youth is dangerous given most have not developed the skills to reason accurately and recognize their long-term interests when presented with short-term gains.

As Matti Häyry and Tuija Takala have noted, individuals can consent to waive their rights to “privacy, confidentiality, non-discrimination, and autonomous decision making,” (Häyry and Takala, 2001, p. 403) which is why WADA and other anti-doping agencies can attain and test blood and urine samples from athletes without creating much controversy, and why many athletes willingly provide the samples. However, when applied to young athletes, who are on their way to becoming autonomous but remain immature, the coercive elements that underlie an athlete's agreement to forgo his or her rights in sport are important but are often ignored. When the only options available to athletes are to adhere to the WADA code or not compete in any WADA-sanctioned events, the consent given by athletes may not be truly voluntarily or freely given. Autonomous athletes can reflect on their choice to waive their rights to medical privacy and freely agree to provide their urine or blood for testing to be eligible to participate, even with recognition that doing otherwise implies guilt and will result in a suspension from competing at the elite level of sport. If Adam Moore is correct that, “controlling who has access to ourselves is an essential part of being a happy and free person” (Moore, 2000, p. 105) then serious ethical implications arise when requiring young athletes to participate in the doping control process. A parent or guardian could counter this concern noting that they can provide consent on behalf of their child to allow anti-doping officials to test their child's urine or blood. The expected assent children must provide alongside their legal guardian's consent to participate in research is often not a focus (Kopelman, 2000).

The utilitarian argument that the rewards of social justice outweigh the costs and consequences of potential privacy and autonomy violations can be very persuasive (Farrelly, 2002). Some young athletes and their legal guardians may conclude that clean sport offsets the violations of privacy that the doping control system creates. For an athlete committed to excellence, taking part in the anti-doping system can be a mere inconvenience or a necessary step to moving forward in their athletic pursuits. But this non-critical approach may stem from a culture of obedience that discourages critical self-reflection and contemplation of one's values and beliefs. Many, but not all, high performance athletes are taught over their many years of intensive training to follow the orders of their coaches and sport-governing bodies and to not question the rules. A young athlete in this category might become accustomed to adhering to rules without first engaging in critical self-reflection or considering the implications of his or her actions outside of the sporting world. Dworkin's second order reflections are likely rarely utilized.

Former WADA director Richard Pound explains how the rules work in sport, and why impartiality and consistency are essential for fairness. Regarding the need for the anti-doping system, Pound reflected in 2004, in a compelling statement no longer available on WADA's website:

[Sport] is governed by rules that, however artificial or arbitrary they may be, are freely accepted by the participants. Why a race is 100 or 200 or 1,500 meters does not really matter. Nor does the weight of a shot or a discuss [sic], the number of members on a team, or specifications regarding equipment. Those are the agreed-upon rules. Period. Sport involves even more freedom of choice than participation in society. If you do not agree with the rules in sport, you are entirely free to opt-out, unlike your ability to opt-out of the legal framework of society. But if you do participate, you must accept the rules. You are not entitled to use a 10-pound shot instead of the 16-pound shot used by your fellow competitors. You are not entitled to start the race before the other competitors, just because you may be a bit slower than they are.^1^

The anti-doping system only exists because people (presumably athletes, sponsors, and fans) want doping-free sport, and athletes can agree to participate for any number of positive or negative reasons: because they value clean sport, because they do not want to get caught, because they do not want to risk damaging their reputation, because they believe their doping will go undetected, and so on.

Coercion can affect young athletes in many ways. The power of coaches, parents, and the athlete entourage can push reluctant youth to use performance-enhancing substances or methods in order the please their mentors, or because they lack support in saying no, as a “coercive environment can inhibit an athlete's autonomous choice to reject the use of performance-enhancing substances” (Miah, 2005, p. 875). The consent that athletes give to anti-doping agencies to have their blood and urine analyzed is not without coercion in the majority of cases (Munthe, 2005). When uncoerced autonomous adults opt to waive their right to privacy and voluntarily accept the rules of sport in order to participate, the resulting restriction of freedom is not a limitation of privacy or autonomy.

Testing is necessary to ensure compliance with the anti-doping rules, but serious ethical concerns arise regarding young athletes' ability to consent if the detection protocol infringes their rights to privacy and autonomy. Pound's argument that athletes can opt out of participating if they do not agree with the rules does not address the objection that children cannot consent to undergo doping control, particularly when we recognize the known pressures that young athletes face and the considerable coercion that coaches and the entourage may be exerting. It is uncertain at what age any individual develops the capacity to understand the implications and potential lifelong health and reputational damage that doping may create. It is equally unclear if it is possible for a young athlete to decide to participate in the anti-doping system, free of coercion, without undue pressure from parents, coaches, and members of the athlete entourage.

Dworkin's account of autonomy, which rests on a person's capacity to reflect critically on first order preferences and accept or attempt to change them in light of higher-order preferences and values, seems missing in sport. Youth athletes accustomed to following the directions and orders of their coaches, trainers, and parents might find this task close to impossible without plenty of prior education. The culture of obedience demanded in sport seems at odds with critical self-reflection and choosing to accept or change one's actions. Of course, there are numerous athletes who have critically evaluated the pros and cons of adhering to the rules set by WADA, the IOC, and their respective IFs, and then made an informed choice to adhere. Blind adherence, without that level of critical reflection, is problematic given the high stakes pressure and coercion known to occur in sport.

## Conclusion: Age Matters

Age requirements have a long history in sport. At the Ancient Olympic Games, where only boys and men were permitted to participate, rather than require that each participant appear in Olympia with proof of his age, the judges “trusted to their eyes and their common sense, instead, with the aim of preventing blatant mismatches” (Finley and Pleket, 1976, p. 62). Boys' events were restricted to competitors who appeared to be between the ages of 12 and 18, but it is possible that tall boys who had not yet turned 12 years of age competed as well. Judges could use their discretion in moving up a well-developed boy to compete in the men's competition if he looked strong enough to contend against the older participants (Finley and Pleket, 1976). Currently, at the Olympic level, the rules stipulated in the Olympic Charter allow each IF to impose age restrictions. Specifically, Rule 42, “Age Limit,” demonstrates that the IOC acknowledges that age matters, stating: “There may be no age limit for competitors in the Olympic Games other than as prescribed in the competition rules of an IF as approved by the IOC Executive Board” (International Olympic Committee, 2021, p. 81). While the IOC does not set age limits on participation as an eligibility rule, responsibility is handed down to the IFs to decide if competitors' age matters. Some IFs have decided that age matters and impose minimum age restrictions for Olympic participation, varying from 13 years of age set by fencing IF and 14 by the IFs for taekwondo and bobsleigh, to 17 years of age set by the IFs for wrestling, cycling, and weightlifting, and 20 years of age for the endurance athletics events governed by World Athletics. The vast discrepancy among disciplines allows 14-year-old divers and bobsled athletes to plunge headfirst into water from heights of 10 meters, or hurl down an ice track wearing minimal protective gear, but not participate in relatively less-risky disciplines. For the young athletes excluded from participating because they do not meet a minimum age limit, the inconsistencies among the rules can seem both arbitrary and unfair (Teetzel, 2010). What is relevant here is that IFs can and do implement minimum age requirements, seemingly in recognition that a certain degree of growth and maturity is needed to compete safely.

The IOC's introduction of the YOG as “a multi-sport, cultural and educational event for young people and driven by young people” (International Olympic Committee, 2007, p. 3) highlights the organization's recognition that age matters in sport. Each IF participating in the Youth Olympic Games sets the age range for eligibility (within the parameter that all competitors at the YOG now must be a minimum of 15 and maximum of 18 years old). As a result of these rules, some youth athletes are eligible to compete at both the YOG and the Olympics, while other young high-performance athletes are eligible for only one or the other, or neither.

There are several good reasons for age restrictions in sport, most of which focus on avoiding early specialization, minimizing the risks of major injuries, and allowing youth athletes to enjoy their youth without undue pressure to excel athletically (Dixon, 2007). These reasons seem equally applicable for determining different levels of culpability when young people dope. The strict liability approaches that are part of the anti-doping movement directly contradict how other spheres recognize the importance of applying age restrictions to protect child and youth athletes.

From exploring the ethical question of whether youth can comprehend fully the implications of taking a banned substance or providing a doping control sample, particularly if coercion or legal guardian pressure is involved, from a philosophical perspective the current system seems unjust. Young athletes have not matured legally or morally, and therefore they should not be subjected to the same punishment and consequences as autonomous adults found to have committed an anti-doping rule violation. What a youth doping sanction would look like remains to be seen, and we must recognize that if there were to be different consequences for doping based on age, then child athletes would be ripe for even further exploitation by unethical coaches and sports clubs. However, a first step is advocating that WADA and the Court of Arbitration for Sport more readily consider an athlete's age and competency when applying the rules that an anti-doping rule violation trigger. Just as IFs are given leeway to choose which age of athletes compete at the YOG, more space to recognize age as a variable in determining the consequences or sanctions after assessing if an anti-doping rule violation has occurred seems warranted.

## Author Contributions

The author confirms being the sole contributor of this work and has approved it for publication.

## Conflict of Interest

The author declares that the research was conducted in the absence of any commercial or financial relationships that could be construed as a potential conflict of interest.

## Publisher's Note

All claims expressed in this article are solely those of the authors and do not necessarily represent those of their affiliated organizations, or those of the publisher, the editors and the reviewers. Any product that may be evaluated in this article, or claim that may be made by its manufacturer, is not guaranteed or endorsed by the publisher.

## Footnotes

^1^This passage was published on the World Anti-Doping Agency website under the title “Remarks by WADA President Richard W. Pound at AAAS annual meeting.” The link to the page is no longer active but was previously available at http://www.wada-ama.org/en/t3.asp?p=41275&x~=1&a=88937.
